# Supplementary material for: An assessment of risk factors for contracting rabies among dog bite cases recorded in Ward 30, Murewa district, Zimbabwe
Source: PLoS Negl Trop Dis. 2021 Mar 31;15(3):e0009305. doi: 10.1371/journal.pntd.0009305 (PMC8691859; doi:10.1371/journal.pntd.0009305)
Supplement: S1 Text — (DOCX) [file pntd.0009305.s001.docx]

| SUMMARY OF VARIABLES |  |  |
| --- | --- | --- |
| VARIABLE | STRATA | SPECIFICS |
| Knowledge about rabies | Animal reservoir in Zimbabwe | Jackal |
|  | Signs and symptoms | Biting without provocation; Agitated behaviour; Growling; Foaming at the mouth; Refusal of food |
|  | Methods of transmission | Biting, scratching and licking of open wound |
|  | Methods of prevention | Dog vaccination; human vaccination |
|  | Source of rabies information | Health officials; television; newspaper; radio; internet; other |
| Practices regarding rabies | Time taken to seek medical attention | Immediately; within a week; within a month |
|  | Place of treatment seeking | Nearest health facility; native/traditional healers |
|  | Dog ownership | Owned or not owned |
|  | Vaccination status of owned dogs | Vaccinated or not vaccinated |
| Dog ownership | Ownership |  |
|  | Non ownership |  |
| Dog bite hot spots | Geographical location |  |
| Spatial distribution of jackals/ jackal presence | Geographical location in relation to rabies cases |  |
