# Supplementary material for: An assessment of risk factors for contracting rabies among dog bite cases recorded in Ward 30, Murewa district, Zimbabwe
Source: PLoS Negl Trop Dis. 2021 Mar 31;15(3):e0009305. doi: 10.1371/journal.pntd.0009305 (PMC8691859; doi:10.1371/journal.pntd.0009305)
Supplement: Attachment — Submitted filename: Reviewer comments on manuscript PNTD - 20- 00750.docx [file pntd.0009305.s002.docx]

**Comment**

*Very good and relevant paper addressing extremely important risk factors for the transmission of an infectious & notifiable disease in Zimbabwe. The findings should be disseminated through the publication. However, the authors can further improve the paper in the following dimensions:*

1. In the abstract (first sentence) the word Rabies should be written in lowercase.
2. Please indicate in the abstract whether the 4 rabies deaths were of laboratory confirmed cases or suspected cases.
3. In the introduction section (last paragraph), “The present study sought to assess risk factors for contracting rabies in Ward 30 of Murewa District. The main aim of this study was to assess risk factors for contracting assessing rabies through assessing knowledge levels, determining practices that may expose people to mapping rabies, mapping dog bite hot spots, determining the vaccination status of owned dogs and determining the spatial distribution of jackals and dogs in relation to rabies cases.’’
(a) The first sentence (study aim) is redundant and should be deleted as it is a repetition of statement below it (study aim).
(b) Were there other aims for this study? If yes state them. If no delete the word “main”.
4. “A pretested interviewer-administered questionnaire designed using guidance from previous studies [35]...”
(a) Please at add least one more study as your statement refers to “studies”
(b) Describe the contents of the questionnaire used or provide an appendix of the questionnaire used.
(c) In which language was the questionnaire administered? If translated, which method did you use to translate the questionnaire? How many researchers participated in this stage? Were they native English speakers or native *Chi*Shona speakers? Reliability of the translated questionnaire (if translated)?
(d) How much time did it take to administer each questionnaire?
(e) Perhaps replace “pretested” with “piloted”
(f) How big the population of the pilot study? What were the pilot study’s results (in brief)? What improvements were made to the questionnaire after piloting it?
5. The discussion section could benefit from some improvements. It’s mainly based on comparisons with other papers. I think the manuscript has potential to discuss regulation requirements, recommendations, etc. For example, although rabies is a notifiable infectious disease in terms Zimbabwe’s Public Health Act (Chapter 15:17), there are no details of priority One Health measures to focus disease prevention and control efforts. In addition, the manuscript’s recommendations are only summarised in the conclusion section and not discussed in the relevant sections in the “discussion”.
6. Provide some methodological limitations of your study and ways to address the limitations. For example, limitations around the study design, self-reports (questionnaires), etc.
7. Please cite at most three references (perhaps recent ones) to support a given point. Perhaps this may help reduce (to maybe ≤ 40 references) the long list of references (88 references) in the “References” section.
8. Please perform proofreading for the whole manuscript & especially check issues of spacing (e.g. references 4 & 10) and position of the fullstops. Provide title of report/publication for reference number 50.
