# Supplementary material for: An assessment of risk factors for contracting rabies among dog bite cases recorded in Ward 30, Murewa district, Zimbabwe
Source: PLoS Negl Trop Dis. 2021 Mar 31;15(3):e0009305. doi: 10.1371/journal.pntd.0009305 (PMC8691859; doi:10.1371/journal.pntd.0009305)
Supplement: Attachment — Submitted filename: Review_16ago2020.docx [file pntd.0009305.s003.docx]

**PLOS Neglected Tropical Diseases**

**An assessment of risk factors for contracting rabies in Ward 30, Murewa District, Zimbabwe**

**Abstract and author summary**

- I assume it is knowledge about rabies, but abstract and author summary do not say so.

**Introduction**

- I would suggest dividing introduction into separate paragraphs to make it more readable.

**Methods**

***Ethics statement***

- Should be mentioned if consent was verbal or written and all actions taken to protect confidentiality of the participants.
- If there is an IRB, should be mentioned.

***Study area***

- Increase font size and size of the crossed in figure 1 would help the reader. Also, in the same figure I can't find the difference between Ward 30 Murehwa and Murehwa Wards nor understand the representation of yellow line. I would recommend increase width of polygon lines and re-arrange arrows in that figure.
- How were categorised high, medium or low-density areas? How dense is high density residential area?

***Methods***

- There is no mention of sample size or sampling methodology in each strata within Ward 30.
- Population could be described better because it is not clear if only households with rabies cases were interviewed or houses with bitten people or houses in general.
- Interviewees were the bitten people or any other people of their families?
- Methods does not specify if both women and men are being interviewed.
- Snowball sampling for dogs and jackals could be explained with more detail to help the reader to understand this procedure.
- It would help to mention how spoor and scat of jackals can be differentiated from the dogs'.
- Surveys were conducted in paper or through cell phones or other elements? This would have implications for the data cleanness.
- Knowledge and practices topics covered in the survey and overall content should be mentioned in methods.

***Statistical analysis***

- It is still needed to mention procedures for practice questions in the questionnaire.
- Procedure about buffer for jackals should be explained with more detail and the use of 12 km is not clear enough.
- How were created hotspots for dogs and jackals?

**Results**

- In methods says that only > 18-year-old are interviewed but in table 1 appear younger people as participants. Participants and interviewees are the same or not?
- Table 1 would look better with consistency about the number of decimals used.
- Table 1 is about 191 participants but in text says that 195 were located.
- Knowledge and practices are referred only as knowledge in the analysis. It is not clear how knowledge of rabies is categorised, so I am not sure about the meaning of "high knowledge level of rabies".
- How was measure time taken to seek treatment and this variable was categorised or not? What was immediately in this study?
- Figure 2 Panel D should work look better in the same dimensions of the other 3 panels.
- Not sure the difference between the number 938 bite cases and 238 bite cases.
- There is no figure 4.
- I recommend including a table for associations and RR and interest variables.
- Were found association with any demographic variable or seeking medical attention?

**Discussion**

- There were 938 dog bite cases, but they were only 263 enrolled and 195 located. What happened with the other 650 bite cases? Why they were not included?

**Other comments**

- Font size in figures and their legends would help the readers.
- I recommend being careful with spaces after dots and some words throughout the document and format of numbers.
