# Supplementary material for: An assessment of risk factors for contracting rabies among dog bite cases recorded in Ward 30, Murewa district, Zimbabwe
Source: PLoS Negl Trop Dis. 2021 Mar 31;15(3):e0009305. doi: 10.1371/journal.pntd.0009305 (PMC8691859; doi:10.1371/journal.pntd.0009305)
Supplement: Attachment — Submitted filename: Response to Reviewers 4 December for submission.docx [file pntd.0009305.s004.docx]

| **GENERAL COMMENTS** |  |
| --- | --- |
| COMMENT/CORRECTION | RESPONSE |
| The methods section could use more clarity.  The methods state that “one family member from each household that had a rabies case was selected for a proxy interview using a questionnaire”.  Were these households that had human rabies cases? | The total number of study participants was 195 including 4 relatives of the human rabies cases.  CORRECTED ON PAGE 6 |
| Elsewhere, the authors state that there were 4 rabies positive cases.  So does this mean that only 4 households were surveyed?  Where then does the 191 sample size come from? Please clarify. | A total of 263dog bite cases were obtained from the Murewa hospital register in Ward 30.This number included 4 human rabies cases. Seventy two dog bite cases out of the 263 could not be reached for interview. The total number of study participants was thus 195. The 195 participants included relatives of the 4 deceased rabies cases  CORRECTED ON PAGE 6 |
| Some terms that were used were also confusing and inconsistently used (e.g., respondent, participant). | CORRECTED ON PAGES 1;3;8;9;15;18; Corrections have been made and the word participants is used throughout the document |
| In the results, the authors talk about 263 respondents.  Where does this number come from? Only 191 were able to respond to the questionnaire, so the 263 individuals should not be called respondents but perhaps ‘enrollees’. How were they enrolled? | Changed 263 respondents to 263 enrolees  The enrolees were recruited by virtue of them being recorded as dog bite cases in the Murewa hospital register for the period January 2017 to July 2018 and all cases recorded during this period were enrolled for the study  CORRECTED ON PAGE 8 and page 6 |
| What questions were asked on the questionnaire?  A table of results from these questions would help. | The questionnaire solicited for data on the transmission of rabies, signs and symptoms and preventive measures. It also sought for information on dog ownership, vaccination status of dogs, dog bite hot spots and presents of jackals.  CORRECTED ON PAGE 6-7  TABLE OF RESULTS FOR THE QUESTIONS IS PRESENTED ON PAGE 10 |
| Understanding how the people surveyed were selected is critical for readers to understand the implications of these results. | All dog bite cases recorded in the Murewa hospital register were enrolled for the study. These included relatives of the 4 human rabies cases  CORRECTED ON PAGE 6 |
| The authors state that “dog owners were more likely to contract rabies as compared to non-dog owners”. This makes is sound like surveys were conducted on a cross section of people that include dog owners and non-dog owners and households that had rabies cases and those that did not.  The sampling that was described does not make it clear who was surveyed and therefore it is not possible to have confidence in this statement. | Among the interviewed dog bite cases and rabies cases, there were dog owners and non-dog owners. Dog ownership was assessed from these people. 191 dog bite cases and 4 rabies cases (proxy interviews) were surveyed.  CORRECTED ON PAGE 6  Dog bite cases that owned dogs were 150 which are 76.92% of the respondents. This is derived from the table showing responses to the questionnaire. This result proved to be statistically significant  CORRECTED ON PAGE 9 AND 10 |
| In the Limitation section of the discussion the authors mention that the 263 mentioned are dog bite cases.  That would mean that the implications for these results can only be applied to people who have been bitten by a dog, this needs to be stated clearly.  Also, in the author summary it is mentioned there are 938 dog bites, how does that relate to the 263 mentioned here? | DONE CORRECTED ON PAGE 18  The 938 dog bites are the Murewa district total. The 263 cases are dog bites from Ward 30, which is one of the 30 wards in Murewa district.  CORRECTED ON PAGE 1. |

| **Detailed comments** |  |
| --- | --- |
| AUTHOR SUMMARY: |  |
| Include Zimbabwe in the description of the study area in the author summary. | CORRECTED ON PAGE 1 |
| It is surprising you are able to get a significant result in a difference in proportions when you only have 4 positive cases.  Please describe this more clearly. | Sample size is 195 dog bite cases among which are 4 human rabies cases.  EXPLAINED ON PAGE |
| INTRODUCTION |  |
| Rabies has no cure, but it is preventable with prophylaxis and this should be mentioned. | CORRECTED ON PAGE 2 |
| METHODS |  |
| Change “It is fairly reliable” to “The rainfall is fairly reliable” | CORRECTED ON PAGE 4 |
| Describe what the Likert Scale does and why it is used. | CORRECTED ON PAGE 7 |
| Provide more detail on the sampling approaches, not just naming the methods.  What is the population from which each sample is taken (who could be selected)?  How many samples are taken? Be clear about the different target populations (all humans, humans who reported dog bites and went to the hospital, etc.) | A total of 263dog bite cases were obtained from the Murewa hospital register in Ward 30.This number included 4 human rabies cases. All the dog bite cases that reported to Murewa hospital between 2017 and 2018 were enrolled for the study. Seventy two dog bite cases out of the 263 could not be reached for interview. The total number of study participants was thus 195. The 195 participants included relatives of the 4 deceased rabies cases  The enrolees were recruited by virtue of them being recorded as dog bite cases in the Murewa hospital register for the period January 2017 to July 2018 and all cases recorded during this period were enrolled for the study  CORRECTED ON PAGE 6 and page 8 |
| RESULTS |  |
| Provide the sample sizes along with the proportions for result. | Corrected by adding results from questionnaires Table 2. Page 10 |
| A table of the questionnaire results would be useful, similar to Table 1. | CORRECTED ON PAGE 10. Table added |
| Provide the numerators and denominators for the tests on the likelihood of contracting rabies. | APPENDIX 3 added |
| LIMITATIONS |  |
| Not all dog bite cases could be reached and there was GPS uncertainty is in the same sentence suggesting these are related points, why? | CORECTED ON PAGE 18 |
| Why do you believe the conclusions can be applied to all of Murewa District?  This is not clear. | Sentence removed  CORRECTED ON PAGE 17 |
| CONCLUSIONS |  |
| Can you quantify the risks based on the spatial maps produced? Figures: | The answer is no. Not with the data we had in this study. While it may be possible and indeed valuable to quantify the risk spatially, this may be easier/ possible in instances were grid/ raster data is used for each of the factors under study. In this study however, the nature of data that we used was vector format and as such in spatial terms, we can only visualise the overlaps and identify areas of risk and using expert knowledge and literature, infer which factors under analysis could be risk factors (without determining the magnitude of that risk). In other words, our goal was to identify (spatially) potential risk factors without looking at magnitude over and above the analysis limitations presented above. |
| Figure 1: the boarder colors for Ward 30 and Murehwa Wards are indistinguishable, please correct this. | Map corrected on page 4 and 5 |
| Figure 1: in the text it suggests that areas are not yet developed and the map is referenced, but that information is not discernable from this map. | Corrected on page 5 and 6. A new map has been provided and explanation given |

WORD DOCUMENT ATTACHMENTS: RESPONSES FROM REVIEWERS

| **Reviewer comments on manuscript PNTD - 20- 00750 Reviewer #1** | |
| --- | --- |
| **Comment**  *Very good and relevant paper addressing extremely important risk factors for the transmission of an infectious & notifiable disease in Zimbabwe. The findings should be disseminated through the publication. However, the authors can further improve the paper in the following dimensions:* | |
| COMMENTS/CORRECTIONS | RESPONSE |
| 1. In the abstract (first sentence) the word Rabies should be written in lowercase. | CORRECTED ON PAGE 1 |
| 2. Please indicate in the abstract whether the 4 rabies deaths were of laboratory confirmed cases or suspected cases. | No laboratory tests were done. The sentence was written as 4 suspected human rabies cases.  CORRECTED ON PAGE |
| 3. In the introduction section (last paragraph), “The present study sought to assess risk factors for contracting rabies in Ward 30 of Murewa District. The main aim of this study was to assess risk factors for contracting assessing rabies through assessing knowledge levels, determining practices that may expose people to mapping rabies, mapping dog bite hot spots, determining the vaccination status of owned dogs and determining the spatial distribution of jackals and dogs in relation to rabies cases.’’ | The last paragraph on the introduction section has been corrected to read: The present study sought to assess risk factors for contracting rabies in Ward 30 of Murewa District through assessment of knowledge, determining practices that may expose people to rabies, mapping dog bite hot spots, determining the vaccination status of owned dogs and determining the spatial distribution of jackals and dogs in relation to dog bite cases.  CORRECTED ON PAGE 3 |
| (a) The first sentence (study aim) is redundant and should be deleted as it is a repetition of statement below it (study aim). | CORRECTED ON PAGE 3  Sentence deleted |
| (b) Were there other aims for this study? If yes state them. If no delete the word “main”. | The whole sentence changed.  CORRECTED ON PAGE 3 |
| 4. “A pretested interviewer-administered questionnaire designed using guidance from previous studies [35]...” | Changed pretested to piloted  CORRECTED ON PAGE 7 |
| (a) Please add at least one more study as your statement refers to “studies” | One more study added CORRECTED ON PAGE 7 |
| (b) Describe the contents of the questionnaire used or provide an appendix of the questionnaire used. | Questionnaire provided as appendix 2  CORRECTED ALSO ON PAGE 8 |
| (c) In which language was the questionnaire administered? If translated, which method did you use to translate the questionnaire? How many researchers participated in this stage? Were they native English speakers or native *Chi*Shona speakers? Reliability of the translated questionnaire (if translated)? | The questionnaire was translated to Chishona the local language. One researcher translated from English to Chishona and the other researcher translated it back to English to check if the meaning remained the same. The questionnaire solicited for data on the transmission of rabies, signs and symptoms and preventive measures. It also sought for information on dog ownership, vaccination status of dogs, dog bite hot spots and presents of jackals.  CORRECTED ON PAGE 7 |
| (d) How much time did it take to administer each questionnaire? | . The questionnaire took an average time of 30 minutes to administer.  CORRECTED ON PAGE 8 |
| (e) Perhaps replace “pretested” with “piloted” | CORRECTED ON PAGE 7 |
| (f) How big the population of the pilot study? What were the pilot study’s results (in brief)? What improvements were made to the questionnaire after piloting it? | A piloted interviewer-administered questionnaire designed using guidance from previous studies [35], [12] was used to assess knowledge and practices pertaining to rabies from dog bite cases including relatives of the rabies cases. The questionnaire was corrected after the piloting which involved 34 participants. Improvements were made on knowledge questions by clearing ambiguities.  CORRECTED ON PAGE 7and 8 |
| 5. The discussion section could benefit from some improvements. It’s mainly based on comparisons with other papers. I think the manuscript has potential to discuss regulation requirements, recommendations, etc. For example, although rabies is a notifiable infectious disease in terms Zimbabwe’s Public Health Act (Chapter 15:17), there are no details of priority One Health measures to focus disease prevention and control efforts. In addition, the manuscript’s recommendations are only summarised in the conclusion section and not discussed in the relevant sections in the “discussion”. | CORRECTED ON PAGE 18, 19 and 20 |
| 6. Provide some methodological limitations of your study and ways to address the limitations. For example, limitations around the study design, self-reports (questionnaires), etc. | It is however important to note that this current study was limited by the fact that only 195 out of 263 dog bite cases were included in the study sample (due to accessibility constraints) and also that proxy interviews were conducted for the 4 rabies cases that were deceased. Furthermore, the GPS device used in this study has a geo-accuracy of ±3 metres which means that the geolocation accuracy of all the mapped data could inherently have this margin of error.  CORRECTED ON PAGE 21 |
| 7. Please cite at most three references (perhaps recent ones) to support a given point. Perhaps this may help reduce (to maybe ≤ 40 references) the long list of references (88 references) in the “References” section. | Some references corrected |
| 8. Please perform proofreading for the whole manuscript & especially check issues of spacing (e.g. references 4 & 10) and position of the fullstops. Provide title of report/publication for reference number 50. | Proof reading done |

| **Review_16ago2020: Revewer #2** | |
| --- | --- |
| COMMENT/CORRECTION | RESPONSE |
| ABSTRACT AND AUTHOR SUMMARY |  |
| - I assume it is knowledge about rabies, but abstract and author summary do not say so. | Corrected on page 1 and 2 |
| INTRODUCTION |  |
| - I would suggest dividing introduction into separate paragraphs to make it more readable. | CORRECTED ON PAGE 2 |
| METHODS: |  |
| Ethics Statement: |  |
| - Should be mentioned if consent was verbal or written and all actions taken to protect confidentiality of the participants. | Consent was obtained from each participant, before conducting the interview. Consent was verbal; participants were asked if they would like to answer a few questions. They were given the choice to take or to decline the interview. To protect confidentiality, participants’ names were not recorded on the questionnaire. In addition, they were assured that their responses would be kept secret. CORRECTED ON PAGE 3/4 |
| - If there is an IRB, should be mentioned. | Approval was also obtained from the National University of Science and Technology.  Corrected on Page 4 |
| Study area: |  |
| - Increase font size and size of the crossed in figure 1 would help the reader. Also, in the same figure I can't find the difference between Ward 30 Murehwa and Murehwa Wards nor understand the representation of yellow line. I would recommend increase width of polygon lines and re-arrange arrows in that figure. | All figures have been edited and the font changed |
| - How were categorised high, medium or low-density areas? How dense is high density residential area? | Explained on page 6  The categorisation is according to the size of residential stands: high density( 200 to 400 square metres), medium density ( 500 to 2000 square metres) and low density( over 200 square metres. CORRECTED ON PAGE 6 |
| Methods: |  |
| - There is no mention of sample size or sampling methodology in each strata within Ward 30. | CORRECTED ON PAGE 6  Stratification was only used to compare risk but not as a sampling strategy |
| - Population could be described better because it is not clear if only households with rabies cases were interviewed or houses with bitten people or houses in general. | Explained, see previous table EXPLAINED/CORRECTED ON PAGE 6/7 |
| - Interviewees were the bitten people or any other people of their families? | Interviewees were bitten people CORRECTED ON PAGE 6. They are described as dog bite cases in the paper except for the relatives of the deceased suspected rabies cases |
| - Methods does not specify if both women and men are being interviewed. | Both men and women were being interviewed CORRECTED ON PAGE 6  This is shown in the demographic characteristics of the participants |
| - Snowball sampling for dogs and jackals could be explained with more detail to help the reader to understand this procedure. | Snowball sampling was used because the total number of the dogs in the ward was not known. Owners of vaccinated dogs were located through records at the Murewa District Veterinary Offices records while those with unvaccinated dogs were located through the snowball sampling approach whereby, a sampled dog owner (regardless of dog vaccination status) was asked to help us locate the nearest dog owner (s) they knew within their respective localities. Corrected on page 7 |
| - It would help to mention how spoor and scat of jackals can be differentiated from the dogs'. | The data collected included location points (in geographic coordinates)of eye-witness accounts/ sightings of jackals. Furthermore, location points of jackal spoor and scat evidence in the area was employed. The jackals poor and scat were identified using the Scat method adopted from Mukherjee [51]. This method involved conventional wildlife, field scat-identification using common characteristics such as morphology (i.e. diameter, length and shape), colour, odour and physical appearance. The location points for spoors were also identified using common wildlife canine characterisations such as heel pad and lobes size, outer and inner toes, outer and inner track size and orientation and claw size. This field activity was carried out with assistance from two animal tracking experts from the Zimbabwe National Parks and Wildlife Authority (ZNPWA). The geographic coordinates of the reported jackal sightings and identified spoors and scat were captured using a  CORRECTED ON PAGE 7 |
| - Surveys were conducted in paper or through cell phones or other elements? This would have implications for the data cleanness. | Surveys were done using printed questionnaires (for interviews with humans); for jackal presence, use of a GPS device for dogs and jackals  CORRECTED ON PAGE 6 |
| - Knowledge and practices topics covered in the survey and overall content should be mentioned in methods. | Knowledge topics were to do with the animal reservoir of rabies in Zimbabwe, signs and symptoms, methods of transmission of rabies, prevention of rabies, treatment seeking following a dog bite and rabies vaccination in dogs. Practice topics encompassed time taken to seek treatment following a dog bite, where to seek treatment, dog ownership and vaccination status of owned dogs. The overall content also included demographic characteristics, dog bite hotspots and spatial distribution of jackals in relation to rabies cases.  CORRECTED ON PAGE 7 |
| Statistical analysis |  |
| - It is still needed to mention procedures for practice questions in the questionnaire. | the procedure for analysing practice questions was conducting of chi square tests and calculation of risk ratio |
| - Procedure about buffer for jackals should be explained with more detail and the use of 12 km is not clear enough. | 12km was the known range of jackal travel according to literature and was created to show possible proximity to habitats and domestic dogs. . The Inverse Distance Weighting (IDW) interpolation algorithm [88] within the QGIS spatial toolbox was then used to generate hotspot maps for jackal presence, dog vaccination status and dog bites whilst proximity analysis using the buffering technique was applied on the jackal presence shapefile (using a 12km buffer range for jackals’ travel range [41]). |
| - How were created hotspots for dogs and jackals? | Dog bite cases, vaccination status and jackal presence shapefiles were overlaid over the Open Street Map background layer and different symbology used to distinguish, visualise and map the different aspects under study. The Inverse Distance Weighting (IDW) interpolation algorithm [88] within the QGIS spatial toolbox was then used to generate hotspot maps for jackal presence, dog vaccination status and dog bites whilst proximity analysis using the buffering technique was applied on the jackal presence shapefile (using a 12km buffer range for jackals’ travel range [41]). |
| RESULTS |  |
| - In methods says that only > 18-year-old are interviewed but in table 1 appear younger people as participants. Participants and interviewees are the same or not? | Participants under 18 were interviewed using ascent if they were dog bite cases. The >18 years restriction was for selected relatives of the deceased human rabies cases  CORRECTED ON PAGE 6  The word participants has replaced interviewees throughout the paper |
| - Table 1 would look better with consistency about the number of decimals used. | Number of decimals corrected |
| - Table 1 is about 191 participants but in text says that 195 were located. | CORRECTED ON PAGE 10 |
| - Knowledge and practices are referred only as knowledge in the analysis. It is not clear how knowledge of rabies is categorised, so I am not sure about the meaning of "high knowledge level of rabies". | Knowledge was categorised according to an adapted scoring system which was based on number of marks scored (Memon *et al*, 2014). So high knowledge level was a score of 52-56 points, medium level was 29-51 points and low level was 14-28 points  CORRECTED ON PAGE 6/7 |
| - How was measure time taken to seek treatment and this variable was categorised or not? What was immediately in this study? | Immediately (within an hour on the same day), Within a week , Within a month |
| - Figure 2 Panel D should work look better in the same dimensions of the other 3 panels. | All figures have been corrected |
| - Not sure the difference between the number 938 bite cases and 238 bite cases. | Explained on page 1. Murewa district recorded 938 dog bite cases and 4suspected rabies deaths between January 2017 and July 2018, overshooting the threshold of zero rabies cases. Of the 938 cases reported in the district, 263 were reported in Ward 30 and these included all the 4suspected rabies deaths reported in the district. This necessitated a study to assess ri |
| - There is no figure 4. | The figures were re numbered |
| - I recommend including a table for associations and RR and interest variables. | Appendix 3 |
| - Were found association with any demographic variable or seeking medical attention? | No demographic variable was assessed for association with seeking medical attention. |
| DISCUSSION |  |
| - There were 938 dog bite cases, but they were only 263 enrolled and 195 located. What happened with the other 650 bite cases? Why they were not included? | They were not included because they were not from the study area, ward 30. 938 cases were for the whole district and 263 were from Ward 30 which is the study area. 68 cases out of the 263 could not be found for interview. The remaining 195 participants were interviewed.  CLARIFIED ON PAGE 6 and 9 |
| OTHER COMMENTS |  |
| - Font size in figures and their legends would help the readers. | All figures were redone and the font changed |
| - I recommend being careful with spaces after dots and some words throughout the document and format of numbers. | The whole document has been revised |
