# Supplementary material for: An assessment of risk factors for contracting rabies among dog bite cases recorded in Ward 30, Murewa district, Zimbabwe
Source: PLoS Negl Trop Dis. 2021 Mar 31;15(3):e0009305. doi: 10.1371/journal.pntd.0009305 (PMC8691859; doi:10.1371/journal.pntd.0009305)
Supplement: Attachment — Submitted filename: Response to reviwers comments 2021 FINAL.docx [file pntd.0009305.s005.docx]

| **COMMENT** | **RESPONSE** |
| --- | --- |
| Be consistent and clear throughout when referring to ‘cases’, be specific as to if you mean dog bite cases, suspected rabies positive cases, or something else. | This has been corrected on pages 1; 3; 6; 9 |
| In the abstract, define “the High Density Cluster”. | The correction has been made in the abstract. |
| In the study area section, what are “Growth Points” and why is this term capitalized? | Corrections have been made in the study area section |
| In Table 2, be clearer in the table caption or headers as to what the number column represents (e.g., number of people who identified dogs as being a rabies reservoir). | HEADERS ON TABLE 2 WERE CORRECTED ON PAGE 10-12 |
| In Table 2, how are there 150 people who owned dogs, then in the next question only 49 people responded if their dog was vaccinated or not.  146 people having no pets does not make sense with 150 saying they own dogs.  Please clarify. | The error has been corrected on page 11-12 |
| In Table 3, give an example of how the questions were worded.  The columns of ‘rabies’ and ‘no rabies’ are unclear.  Were all people in the study asked these questions? | Corrected on page 12 |
| The inclusion of Table 3 is helpful.  The contingency tables within it are useful.  However, with only 4 suspected positive rabies cases the sample sizes are not sufficient to conduct a chi-square tests, see McHugh 2013.  Therefore, the chi-square analyses should be removed from the manuscript.  However, I think the value of the information being shown is still there even without these tests.  It is still reasonable to look at odds ratios or risk ratios, as these are descriptive statistics and not statistical tests that have sample size requirements.    McHugh, M. L. 2013. The chi-square test of independence. Biochemia medica 23:143-149. | The Chi square tests have been removed and the odds ratios/risk ratios have been retained, page 1;8; 12;13  The contingency tables have been retained but without the chi-square tests. |

**RESPONSE TO REVIEWERS COMMENTS**
